# Long‐Term Results of Anatomic Stemless Shoulder Prosthesis in Patients With Primary Osteoarthritis

**DOI:** 10.1111/os.70356

**Published:** 2026-06-05

**Authors:** Kevin Knappe, Franziska Becker, Raphael Trefzer, Mustafa Hariri, Anna‐Katharina Nolte, Matthias Bülhoff

**Affiliations:** ^1^ Department of Orthopaedic Surgery Heidelberg University Hospital Heidelberg Germany; ^2^ Department of Traumatology, Hand Surgery and Sports Medicine ViDia Clinics Karlsruhe Germany

**Keywords:** long‐term outcome, shoulder arthroplasty, shoulder osteoarthritis, stemless

## Abstract

**Objectives:**

Shaft‐anchored prostheses have long been the gold standard in shoulder arthroplasty, but there is a growing trend toward bone‐preserving, stemless designs. Despite promising short‐ and midterm results, long‐term data remain limited. This study aims to report the long‐term outcomes of a stemless anatomic shoulder prosthesis in patients with primary osteoarthritis and seeks to answer the question of whether it is comparable to other prostheses.

**Methods:**

This retrospective single‐center study included 27 shoulders in 24 patients (mean age 75 ± 8.3 years), who were operated from 2009 to 2011. Outcomes assessed were Constant‐Murley score, range of motion, patient satisfaction, revision rate, and radiological findings. Mean follow‐up was 142 ± 12.2 months. Twenty‐one shoulders underwent total shoulder arthroplasty; six hemiarthroplasty. For the analysis of continuous variables, the Wilcoxon test was used following normality testing. Dichotomous variables were evaluated using the chi^2^ test, with a significance level set at *p* < 0.05.

**Results:**

The Constant‐Murley score improved significantly from 23.1 ± 9.4 (27.5% age‐adjusted) preoperatively to 58.8 ± 18.6 (72.3% age‐adjusted) postoperatively (*p* < 0.001). Range of motion increased in flexion (89.5°–137.2°), abduction (70.9°–117.2°), and external rotation (10°–36.8°), all statistically significant. Overall, 85% of patients were (very) satisfied. Four revisions were performed: two with conversion to alternative treatment, two within the same system due to loosening and infection. Ten‐ and 12‐year survival rates were 89% and 85%, respectively. A 30% risk of loosening was observed (per Molé), though no glenoid loosening occurred; humeral loosening was seen in one case.

**Conclusion:**

The clinical, functional, and radiological outcomes of the investigated stemless shoulder arthroplasty system remain satisfactory even in long‐term follow‐up. High patient satisfaction was observed. The data, which are unique in terms of the length of follow‐up, are comparable to those of other stemless anatomic shoulder arthroplasty designs.

## Graphical abstract

Stemless anatomic shoulder prostheses demonstrate sustained improvements in function and range of motion, with high patient satisfaction and low revision rates, even after exceptionally long follow‐up. Outcomes are comparable to other stemless designs, supporting their long‐term effectiveness in primary osteoarthritis.

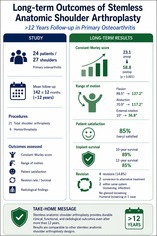

## 1 Introduction

In shoulder arthroplasty, shaft‐anchored prostheses have long represented the gold standard for treating degenerative and post‐traumatic joint diseases. However, concerns regarding humeral bone loss, stress shielding, and the potential complications associated with stemmed implants such as intraoperative humeral fractures that occur at a rate of around 1.5% [1, 2, 3, 4] have led to the development of bone‐preserving, stemless shoulder prostheses [5, 6]. Thesecould show good and similar functional outcomes, complication and revision rates compared to conventional stemmed arthroplasty [2, 7]. Some data even suggest a positive influence on the postoperative range of motion following stemless implantation compared to conventional stemmed prostheses [8]. In addition, estimated blood loss and the mean operative time are significantly lower with a stemless system [9, 10].

Instability, rotator cuff, and glenoid failure are the most common causes of revision surgery [11]. In up to 50%, the glenoid component shows radiographic signs of loosening [11, 12, 13]. Humeral components are therefore rather not the reason for revision but might cause problems in revision surgery, particularly in cases involving long cemented components [14].

Stemmed and stemless shoulder arthroplasty do have the same indications, but there are specific contraindications for stemless prostheses, such as poor metaphyseal bone stock, proximal humeral fractures, large metaphyseal cysts, pseudarthrosis, or osteoporosis [15, 16]. Age itself is no contraindication for stemless shoulder arthroplasty surgery [17, 18, 19].

The Total Evolutive Shoulder System (T.E.S.S.—Biomet/Zimmer, Warsaw, USA) is designed to restore the shoulder joint anatomy by using a stemless humeral component that integrates to the metaphyseal bone [20]. The aim of this study was to evaluate (i) clinical outcomes and implant survival, as well as (ii) radiological findings, following implantation of this stemless shoulder prosthesis in patients with primary osteoarthritis at a single orthopedic center. Midterm results of this patient cohort have been published previously [21].

## 2 Methods

### 2.1 Study Population and Follow‐Up

**FIGURE 1:**
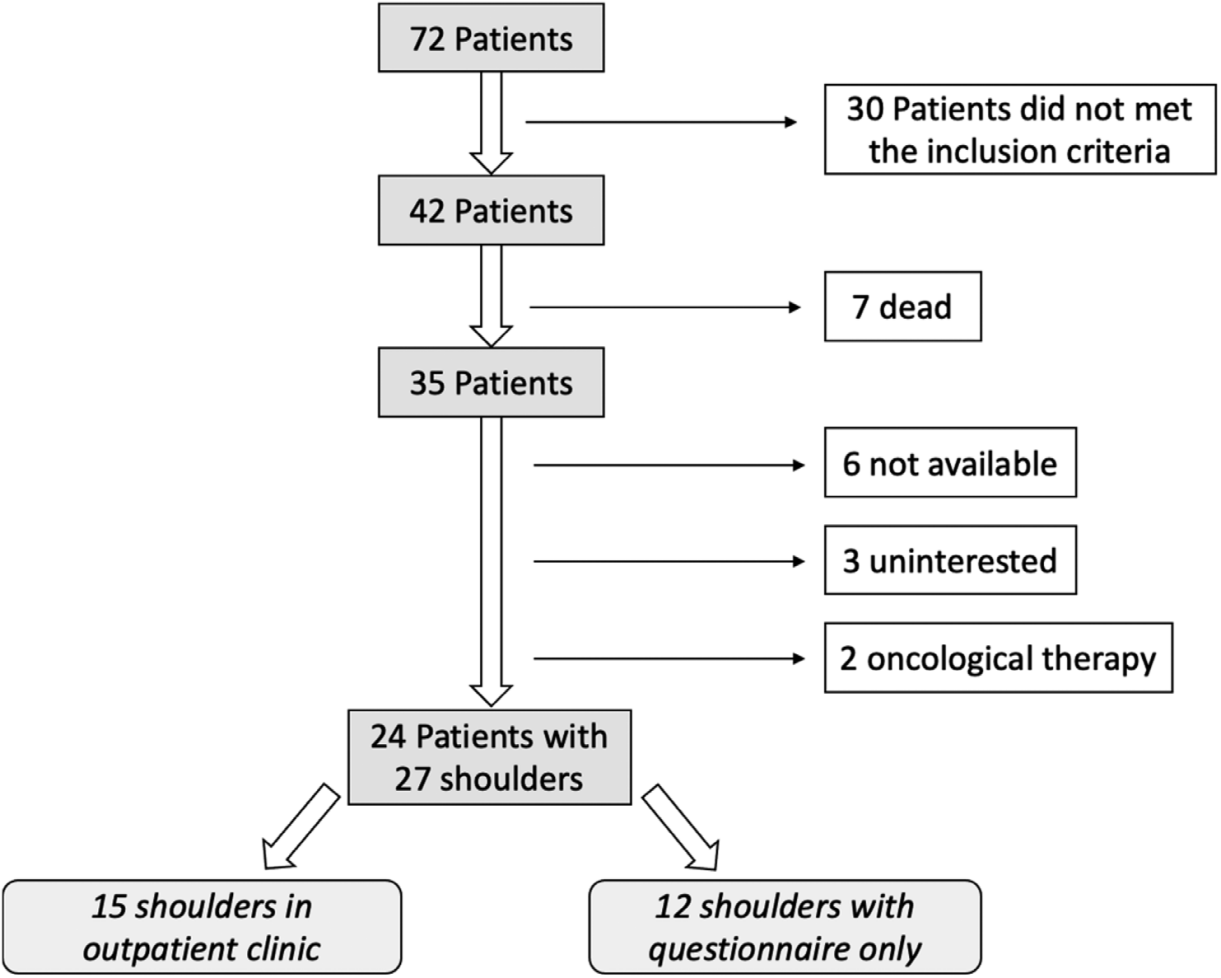
Flowchart of patient recruitment.

A total of 480 shoulder arthroplasties were performed at our institution between 2009 and 2011, of which 72 were treated with the anatomical T.E.S.S. The inclusion criteria for this study were: (a) primary osteoarthritis of the shoulder, (b) an intact rotator cuff, and (c) a minimum follow‐up of 9.5 years. Forty‐two cases met these criteria. Seven died before the follow‐up, with their deaths not being directly related to the surgery performed. Six patients were lost to follow‐up and five declined participation in the study. Consequently, a total of 11 patients were not available for follow‐up. Thus, 24 patients with 27 shoulders could be recruited for final follow‐up (Figure 1). Fifteen shoulders were examined in the outpatient clinic, while 12 were evaluated using a certified questionnaire [22]. In the latter group, strength was assessed using the validated Constant‐based self‐evaluation method as described by Boehm et al. [22].

Both clinical and radiological assessments were conducted. The study received approval from the university's ethics board (board‐application number: S‐305/2007). The clinical evaluation included the Constant‐Murley score, which was collected preoperatively and archived in the clinic's internal database (also adjusted for age and sex), as well as range of motion in flexion, abduction, and external rotation, and patient satisfaction. At the final follow‐up, patients were asked to rate their satisfaction with the shoulder replacement surgery as “very satisfied,” “satisfied,” “undecided,” or “disappointed.”

### 2.2 Surgical Technique and Rehabilitation

The surgical technique was performed following the well described approach of Kadum et al. [23]. After a deltopectoral approach, the rotator interval is divided, the subscapularis muscle is detached, and the capsule is released along the humeral neck to facilitate adequate glenoid exposure, accompanied by osteophyte removal and anterior dislocation of the humeral head for preparation. Glenoid resurfacing involved guidewire placement, controlled drilling, component fixation, and final stabilization, followed by subscapularis tensioning, closure, and postoperative immobilization. Postoperative rehabilitation for subscapularis repair limited passive external rotation to 0° for 6 weeks, with active internal rotation prohibited. Abduction was restricted to ≤ 90° during the first 4 weeks. All exercises were performed under physiotherapeutic supervision and pain‐free. From Week 5, progressively active exercises were allowed, with return to heavy physical activity permitted at 3 months.

### 2.3 Radiographic Evaluation

**FIGURE 2:**
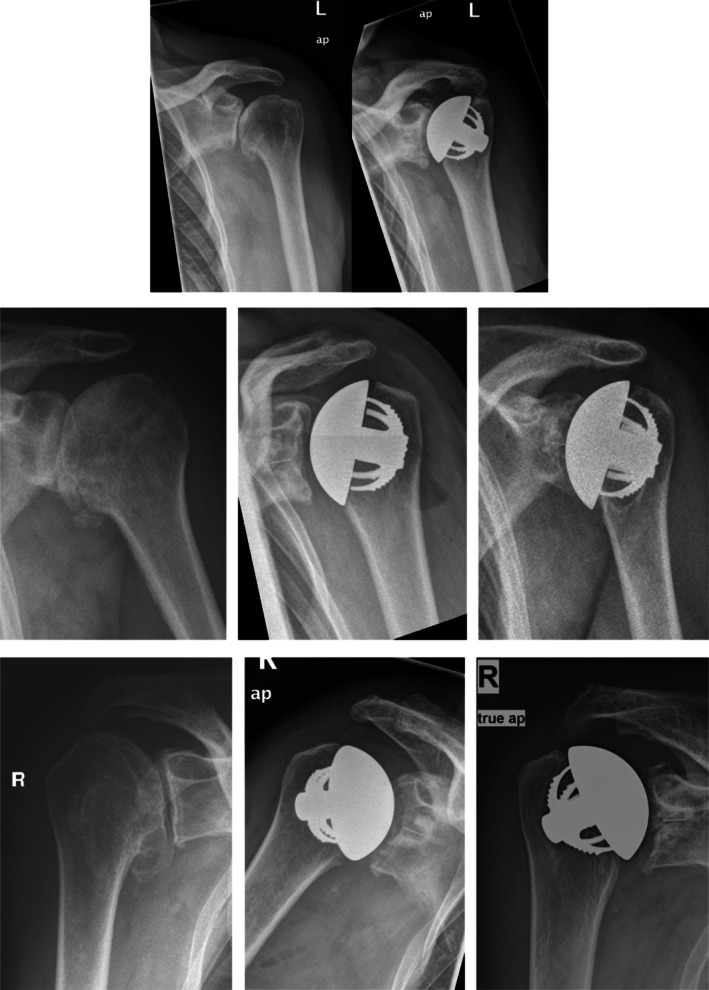
Pre‐ and postoperative radiograph of a left shoulder. Left shoulder of a 68‐year‐old female patient at the time of surgery with primary glenohumeral osteoarthritis. Left: Preoperative. Center: 4 days postoperative. Right: 12 years and 3 months postoperative. Right shoulder of a 61‐year‐old male patient at the time of surgery with primary glenohumeral osteoarthritis. Left: Preoperative. Center: 3 days postoperative. Right: 13 years and 5 months postoperative.

Preoperative imaging included a true anteroposterior (AP) view and an axillary view of the affected shoulder. Magnetic resonance imaging (MRI) was performed to evaluate rotator cuff integrity, and computed tomography (CT) scans were obtained to assess patient‐specific anatomical characteristics and to enable precise preoperative surgical planning for shoulder arthroplasty. At final follow‐up examination, true AP and axillary views were taken (Figure 2). For radiographic evaluation, 15 shoulders were included postoperatively, as part of the cohort was followed up by phone and questionnaires, as previously described.

Radiographs were analyzed for signs of loosening, specifically looking for radiolucent lines around the cemented glenoid component, by two surgeons specialized in shoulder arthroplasty to ensure interobserver consistency. Interobserver discrepancies were resolved by consensus discussion. Although no formal statistical agreement coefficient was calculated, this dual independent assessment was used to ensure consistency of radiographic interpretation. The analysis followed the classification system by Molé et al. [24]. Both the AP and axillary views were assessed for radiolucent lines, and points were assigned based on their presence. The points from both views were then summed. A total of up to six points indicated no risk of loosening, 7 to 12 points suggested a risk of glenoid loosening, and a total of more than 12 points indicated a loose glenoid component. The detailed protocol has been previously described [12]. Additionally, the anatomical restoration of the proximal glenohumeral joint was assessed by measuring of the lateral offset. The glenoid morphology of the shoulders included in the study was assessed based on the Walch classification [25].

### 2.4 Statistical Evaluation

For continuous variables, the Wilcoxon test was used for significance testing after assessing for normal distribution. Dichotomous variables, such as gender or handedness, were analyzed using the Chi‐squared test. All data were analyzed using IBM SPSS Statistics (IBM Corp. Released 2023. IBM SPSS Statistics for Macintosh, Version 29.0.2.0 IBM Corp., Armonk, NY, USA) with a significance level of *p* < 0.05.

ChatGPT (GPT‐5, OpenAI, 2025) was used to improve spelling, grammar, and general editing in accordance with the journal's guidelines.

## 3 Results

### 3.1 Study Population

A total of 24 patients (27 shoulders) were included in the study. The mean age at the time of surgery was 62 years (range 47–75 years). At a mean follow‐up of 142 ± 12.2 months (116–158 months) postoperatively, the patients had a mean age of 75 years (±8.3 years, range 60–93 years). The cohort consisted of 10 female and 14 male patients, with 12 left and 15 right shoulders affected. In 14 cases, the dominant side was affected, and in 13 cases, the non‐dominant side. Four shoulders had undergone previous surgery prior to the arthroplasty intervention. In three cases, a conversion from a CUP prosthesis to a total shoulder arthroplasty (TSA) was performed, while in one case, a hemiarthroplasty was converted to a TSA. In all cases, preoperative assessment confirmed intact rotator cuff integrity and preserved joint centering, permitting uncomplicated conversion to an anatomic TSA.

A total of 21 total shoulder arthroplasties and six hemiarthroplasties were implanted and followed up. A total of eight type A1 glenoids, five type A2 glenoids, three type B1 glenoids, seven type B2 glenoids, and four type C glenoids were treated surgically. All shoulders treated with a hemiarthroplasty had a type A glenoid.

At the time of follow‐up, 23 of the 27 implanted prostheses remained in situ. During the first year after surgery, two shoulders required revision due to loosening and infection. They were reimplanted using the T.E.S.S. system. In the other two revision cases, conversion to a conventional, shaft‐anchored reverse shoulder arthroplasty (RSA) was performed after 9 and 13 years, respectively. In one of those cases, a glenoid erosion occurred following hemiarthroplasty. In the other case, the cause revision remains unknown (Table 1).

**TABLE 1:** Descriptive statistics.

Variable	Value
Age, mean (SD; range; years)	62 (8.1; 48–80)
Gender, female/male	10 (37%)/17 (63%)
Primary osteoarthritis	23 (85%)
Conversion from hemi‐ to total shoulder arthroplasty	4 (15%)
Clinical follow‐up in months, mean (SD; range)	142 (12.2; 116–158)

### 3.2 Clinical Outcomes

**FIGURE 3:**
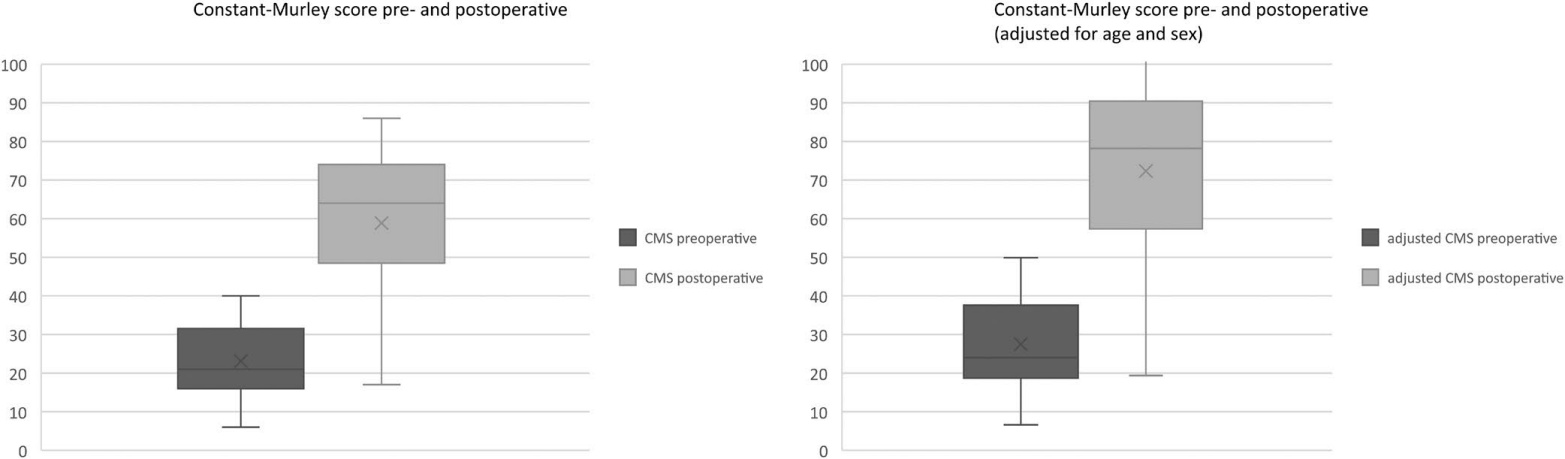
Constant‐Murley score (left) and CMS adjusted for age and sex (right), both pre‐ and postoperation.

Clinical outcome according to the CMS from a preoperative mean of 23.1% (9.4; 6–40) to a postoperative mean of 58.8% (18.6; 17–86) (*p* < 0.001) (Figure 3, left). Similarly, the gender‐adjusted CMS (relative CMS) showed a significant improvement from 27.5% (11.5; 6.6–49.9) to 72.3% (23; 19.4–105.1) (*p* < 0.001) (Figure 3, right). In this context, it should be noted that range of motion measurements were performed actively in accordance with the standardized Constant‐Murley score protocol.

Significant improvements were also observed in the subdomains. At the time of follow‐up, patients reported less pain and fewer limitations in daily activities. In the Constant‐Murley score, a low pain level is associated with higher scores. Additionally, range of motion and strength had significantly improved (Table 2).

**TABLE 2:** Constant‐Murley score subdomains [Mean(SD, range)].

			*p*
	Preoperative	Postoperative/follow‐up	
Pain (max. 15)	2.6 (2.5; 0–5)	12 (4.1; 0–15)	< 0.001
Activities of daily living (max. 20)	6.3 (3.3; 2–11)	15.5 (4.1; 4–20)	< 0.001
Movement (max. 40)	13.3 (7.3; 4–36)	25.5 (7.3; 4–36)	< 0.001
Strength (max. 25)	0.8 (1.6; 0–5)	7.5 (4.9; 1–18)	< 0.001

### 3.3 Radiographic Evaluation

Radiographic analysis included 15 out of 25 shoulders (60%) postoperatively. The evaluation of the X rays was conducted by two experienced shoulder surgeons. According to Molé's evaluation criteria, four shoulders were identified as being at risk for loosening. Additionally, one humeral component showed signs of loosening. No revisions were required in any case. Four shoulders exhibited periarticular ossifications. Three shoulders showed significant cranialization and two signs of stress shielding. The lateral offset preoperatively was 26.8 mm (3.3; 23.6–34.1) and postoperatively 25.3 mm (2.4; 20–37.4).

### 3.4 Patient Satisfaction

The entire patient cohort (including dropouts) was surveyed regarding their subjective satisfaction with the shoulder prosthesis surgery. The majority of patients reported being “very satisfied” or “satisfied.” Four patients were “undecided” or “disappointed.” Overall, 85% were “very satisfied” or “satisfied” with the outcome. Reasons for being “disappointed” included limited shoulder function and sleep disturbances on the side of the operated shoulder.

## 4 Discussion

In this cohort of 24 patients (27 shoulders) with a mean follow‐up of 12 years, anatomical shoulder arthroplasty demonstrated significant and sustained clinical improvement. The CMS increased from 23.1 preoperatively to 58.8 postoperatively (*p* < 0.001), with parallel improvements in pain, function, range of motion, and strength. Implant survival was high, with 23 of 27 prostheses remaining in situ at final follow‐up. Radiographic changes were observed in a minority of cases but did not necessitate revision. Overall patient satisfaction was high, with 85% reporting being satisfied or very satisfied with the outcome.

This study represents one of the few long‐term investigations with a minimum follow‐up of 9.5 years evaluating stemless anatomical TSA. The aim was to assess long‐term clinical outcomes, implant survival, and radiological findings of the anatomical T.E.S.S. prosthesis.

### 4.1 Advantages and Disadvantages of Anatomic Stemless Shoulder Prosthesis

Stemless anatomic shoulder arthroplasty represents a bone‐conserving alternative to conventional stemmed implants, with several clinically relevant advantages. Because fixation is achieved within the metaphysis rather than the diaphyseal canal, humeral bone stock is preserved, which can simplify potential revision procedures. The avoidance of a diaphyseal stem may also reduce stress shielding phenomena and decrease the risk of periprosthetic fractures.

At the same time, this implant concept is not without limitations. Its success is strongly dependent on sufficient metaphyseal bone quality, which may restrict its indication in elderly patients or in cases of osteoporotic or otherwise compromised proximal humeral bone. Moreover, long‐term outcome data are still comparatively limited when contrasted with established stemmed prosthesis systems. Potential concerns such as metaphyseal loosening or subsidence in selected situations therefore remain relevant. Consequently, careful patient selection is essential (Table 3).

**TABLE 3:** Comparison of the characteristic properties of stemless and stemmed anatomic shoulder arthroplasty.

Feature	Anatomic stemless shoulder arthroplasty	Anatomic stemmed shoulder arthroplasty
Fixation principle	Metaphyseal	Diaphyseal/metapyseal‐diaphyseal
Bone preservation	High	Lower
Intraoperative fracture risk	Lower	Higher
Implant stability	Highly dependent on metaphyseal bone stock	More tolerant to compromised metaphyseal bone due to diaphyseal fixation
Revision potential	Generally favorable (no stem removal)	More complex due to stem extraction
Periprosthetic fracture risk	Lower	Higher

### 4.2 Clinical Outcomes and Implant Survival

Several studies on the stemless prosthesis described in this study (T.E.S.S.) have already reported good midterm outcomes and an improvement in the CMS from 30 up to nearly 80 after three to four years [21, 26]. Results of this study are comparable to the only study reporting long‐term outcomes of the anatomical T.E.S.S. prosthesis [27]. 30 shoulders with a mean follow‐up of 94 months showed an improvement of the CMS from 15 to 68. A survival rate was not specifically reported. But, no other long‐term data on this prosthesis were available at the time of this study. When comparing the results with other stemless shoulder prostheses, similar outcomes are observed in terms of improvement in the CMS with postoperative values ranging between 70 and 80 and long‐term survival rates [28, 29]. However, in the case of the study by Hawi et al., it should be noted that a large number of hemiarthroplasties were implanted, which limits comparability and reduces the significance with regard to the present study.

Magosch et al. showed an estimated 13‐year survivorship rate of 90.1% (Eclipse stemless shoulder prosthesis; Arthrex, Munich, Germany), Martens et al. 91.5% after 118 months (Total Evolutive Shoulder System T.E.S.S.; Biomet, USA) [30, 31]. This argument is contradicted by a survival analysis from the Australian Orthopedic Association's National Joint Replacement Registry published in 2024, which included 3156 implanted stemless shoulder prostheses and reported a revision rate of only 4.4%. However, the mean follow‐up period in this Australian registry‐study was only 3.1 years (±2.3). When looking at 3 years as a reference, the revision rate in the present study decreases to 7.4%. Of the 27 implanted prostheses, 23 remained in situ at the time of follow‐up. Two prostheses required revision within the first postoperative year due to loosening and infection, emphasizing the importance of rigorous postoperative management. Two additional prostheses underwent conversion to RSA after 9 and 13 years, respectively, due to prosthetic failure. The observed revision rate of 14.8% at the end of follow‐up and the 10‐year revision rate of 11.1%, respectively is slightly higher than the range reported in the mentioned studies on mid and long‐term outcomes of anatomical shoulder arthroplasties. This highlights the importance of careful patient selection and precise implantation to optimize outcomes.

### 4.3 Radiological Findings

Radiolucent lines in the region of the cemented glenoid component are a well‐known long‐term radiological finding of stemless shoulder prosthesis that frequently occur in a substantial number of cases [27]. Aibinder et al. also observed in their study of 152 shoulders that stress shielding occurred in a significant number of cases, with a prevalence of 41% after a follow‐up of just two years [32]. In contrast, other studies have reported a lower percentage, with only 7% of cases being affected by stress shielding [33]. In that context, it is important not to confuse the different radiological appearances of stress shielding and radiolucent lines. Stress shielding reduces bone loading, causing bone resorption, while radiolucent lines indicate insufficient osseointegration or micromovement, signaling implant instability. In the present study, radiological evaluation revealed good stability and implant integration postoperatively. According to Molé's criteria, four shoulders were classified as at risk of loosening, and one humeral component exhibited signs of loosening. However, these radiological findings did not correlate with functional impairment or patient dissatisfaction.

### 4.4 Limitations and Strengths

This study has several limitations that must be considered when interpreting the results. The small sample size of 27 shoulders limits the statistical power and generalizability of the findings. Larger cohorts are necessary to validate these outcomes in a broader population. The radiological evaluation was conducted in 15 of 25 shoulders (60%) due to the follow‐up methodology. This limitation may have affected the ability to comprehensively assess radiological changes. Therefore, the reported radiological outcomes and loosening rates must be interpreted with caution, as they may not fully represent the entire study population. Furthermore, the retrospective design of the study introduces a potential for selection bias and incomplete data collection, which could influence the robustness of the conclusions. Finally, the lack of a control group comparing stemless anatomical shoulder prostheses to stemmed prostheses or other stemless models limits the ability to draw direct comparisons and assess the relative advantages or disadvantages of the two approaches. To the best of our knowledge, long‐term results from comparative studies on stemless and stemmed shoulder prostheses are not available. However, both short‐term comparisons and a meta‐analysis by Liu et al. found no significant differences in postoperative CMS or complication rates [9, 10, 34]. These limitations highlight the need for future prospective studies with larger patient populations and controlled comparative designs.

## 5 Conclusion

The clinical and radiological outcomes of the investigated stemless shoulder arthroplasty system remain satisfactory even in the long‐term follow‐up. High patient satisfaction along with significant improvements in clinical function and pain reduction was observed. The results are comparable to those of other stemless shoulder arthroplasty designs, but due to the exceptionally long follow‐up period, they are nearly unique. However, the observed revisions and radiological changes warrant further investigation to optimize biomechanical properties and clinical outcomes of these implants. Our study demonstrates that stemless anatomical shoulder arthroplasty is an effective option for the treatment of end‐stage degenerative shoulder conditions. The results indicate significant improvements in clinical function and pain reduction, with most implants remaining stable over time.

## Author Contributions

**Kevin Knappe:** conceptualization, formal analysis, data curation, project administration, writing – original draft, writing – review and editing. **Franziska Becker:** conceptualization, formal analysis, data curation, project administration, writing – original draft, writing – review and editing. **Raphael Trefzer:** writing – review and editing. **Anna‐Katharina Nolte:** project administration, writing – review and editing. **Mustafa Hariri:** writing – review and editing. **Matthias Bülhoff:** conceptualization, data curation, writing – review and editing.

## Funding

The authors have nothing to report.

## Ethics Statement

The study received approval from the university's ethics board (Board‐application number: S‐305/2007). The study was conducted in accordance with the Declaration of Helsinki (as revised in Brazil in 2013).

## Consent

Informed consent was obtained from all individual participants included in the study. All authors have read and agreed to this version of the manuscript.

## Conflicts of Interest

M.B. is a paid consultant for Stryker (Memphis, TN, USA). A.‐K.N. has received lecture fees from DePuy Synthes (Warsaw, IN, USA). The other authors declare no conflicts of interest.

## Data Availability

The data that support the findings of this study are available from the corresponding author upon reasonable request.
